# Effects of a Diet-Based Weight-Reducing Program with Probiotic Supplementation on Satiety Efficiency, Eating Behaviour Traits, and Psychosocial Behaviours in Obese Individuals

**DOI:** 10.3390/nu9030284

**Published:** 2017-03-15

**Authors:** Marina Sanchez, Christian Darimont, Shirin Panahi, Vicky Drapeau, André Marette, Valerie H. Taylor, Jean Doré, Angelo Tremblay

**Affiliations:** 1 Faculty of Medicine, Department of Kinesiology, Laval University, Quebec, QC G1V 0A6, Canada; marina.sanchez@criucpq.ulaval.ca (M.S.); shirin.panahi1@ulaval.ca (S.P.); jean.dore@kin.ulaval.ca (J.D.); 2 Nestlé Research Center, Department of Metabolic Health, 1000 Lausanne, Switzerland; christian.darimont@rdls.nestle.com; 3 Faculty of Educational Sciences, Department of Physical Education, Laval University, Quebec, QC G1V 0A6, Canada; vicky.drapeau@fse.ulaval.ca; 4 Research Center of the Institut Universitaire de Cardiologie et de Pneumologie de Québec, Quebec, QC G1V 4G5, Canada; andre.marette@criucpq.ulaval.ca; 5 Department of Psychiatry, University of Toronto, Toronto, ON M5S 1B2, Canada; Valerie.Taylor@wchospital.ca

**Keywords:** probiotics, body weight, food intake, appetite sensations, eating behaviors, gut-brain axis

## Abstract

This study evaluated the impact of probiotic supplementation (Lactobacillus rhamnosus CGMCC1.3724 (LPR)) on appetite sensations and eating behaviors in the context of a weight-reducing program. Obese men (*n* = 45) and women (*n* = 60) participated in a double-blind, randomized, placebo-controlled trial that included a 12-week weight loss period (Phase 1) based on moderate energy restriction, followed by 12 weeks of weight maintenance (Phase 2). During the two phases of the program, each subject consumed two capsules per day of either a placebo or a LPR formulation (10 mg of LPR equivalent to 1.6 108 CFU/capsule, 210 mg of oligofructose, and 90 mg of inulin). The LPR supplementation increased weight loss in women that was associated with a greater increase in the fasting desire to eat (*p* = 0.03). On the other hand, satiety efficiency (satiety quotient for desire to eat) at lunch increased (*p* = 0.02), whereas disinhibition (*p* = 0.05) and hunger (*p* = 0.02) scores decreased more in the LPR-treated women, when compared with the female control group. Additionally, the LPR female group displayed a more pronounced decrease in food craving (*p* = 0.05), and a decrease in the Beck Depression Inventory score (*p* = 0.05) that was significantly different from the change noted in the placebo group (*p* = 0.02), as well as a higher score in the Body Esteem Scale questionnaire (*p* = 0.06). In men, significant benefits of LPR on fasting fullness and cognitive restraint were also observed. Taken together, these observations lend support to the hypothesis that the gut-brain axis may impact appetite control and related behaviors in obesity management.

## 1. Introduction

The progress in recent research has allowed a better characterization of the etiology of obesity, by emphasizing the potential contribution of factors not traditionally considered to be involved in variations in energy balance and body composition. Evidence suggests that inadequate food-related behaviors are more predictive of overweight individuals than suboptimal macronutrient diet composition [1]. Experimental and clinical studies have also demonstrated the role of gut microbiota in the regulation of energy balance and occurrence of excess body weight [2,3]. In this regard, it has been suggested that lean and obese humans differ in the composition of their gut microbiota [4], and that prebiotics and probiotics can be used to manipulate the microbiota to prevent body weight gain [5]. Recently, some studies have demonstrated the role and importance of the gut microbiota in modulating the activity of the central nervous system [2]. Evidence suggests that the gut microbiota and brain “talk to each other”, contributing to what is commonly known as the gut-brain axis [6,7]. This bidirectional link allows signals from the brain to influence motility, appetite sensations, secretions, and permeability of the gut. Conversely, some gut messages may influence brain functions involved in the regulation of stress, depression, and anxiety [8].

The idea that microbiota changes are involved in mood, depressive behavior, and stress is supported by several animal studies. Lyte and collaborators [9] demonstrated that the consumption of a pathogenic bacteria such as Campylobacter jejuni results in the activation of the immune system (increased cytokines), which is associated with the development of anxious behavior in mice [9,10]. Furthermore, Gareau et al. [11] showed that the microbiota are essential for the normal cognitive development of mice [11]. Additionally, dietary changes may also explain the communication between the microbiota and brain. A recent study showed that dietary modification is associated with both a positive change in the composition of the microbiota, and a reduction in anxiety-related behaviors [12]. It is also known that some strains of Lactobacillus have the ability to produce neurotransmitters, particularly gamma-aminobutyric acid (GABA) [13], and increase the activity of certain enzymes involved in the metabolism of tryptophan, a precursor of serotonin [14]. GABA and serotonin are known for their positive effects on depression and anxiety [15].

The concept of a gut-brain axis is of particular interest for health professionals treating obesity, since weight loss has been shown to influence feeding behaviors and related variables. In this field, one of the key issues is the effect of a substantial amount of body weight loss, which increases appetite sensations (e.g., hunger and desire to eat) [16,17]. Moreover, our research and clinical experiences have shown that body weight loss in obese individuals who are resistant to losing more body fat may have an increased proneness to depression, as reflected by a higher Beck Depression Inventory score [18,19].

In summary, these observations suggest that substantial body weight loss promotes effects such as an increase in the symptoms of depression, which may lead to the resistance to lose more body fat and, ultimately, favor body weight regain. The literature also highlights the potential beneficial effects of probiotic supplementation on appetite control and related behaviors that ultimately contribute to the sustainability of a weight-reduced obese state. Thus, the objective of this study was to investigate the effects of probiotic supplementation (Lactobacillus rhamnosus CGMCC1.3724) on eating behaviors and mood in obese individuals submitted to a weight-reducing program. We hypothesized that probiotic supplementation can positively influence appetite sensations, mood, and eating behavior traits in obese men and women, under conditions of negative energy balance and subsequent body weight maintenance. In this regard, we have pursued the analysis of relevant data obtained in a study which showed that the supplementation of Lactobacillus rhamnosus CGMCC1.3724 accentuated body weight loss in obese women subjected to dietary supervision and promoted negative energy balance [20].

## 2. Materials and Methods

### 2.1. Protocol

Obese men (*n* = 45) and women (*n* = 60) participated in a 24-week randomized, double-blind, placebo-controlled trial that was aimed at body weight loss (Phase 1) and subsequent weight maintenance (Phase 2). The protocol consisted of two parts: phase 1 was a 12-week weight loss program involving supervised dietary restriction and phase 2 was a 12-week weight maintenance program with supervised dietary habits without restriction. Each participant received a personalized diet plan targeting a 2092 kJ/day (500 kcal/day) energy restriction for the first phase of the program. During phase 2, each participant received a personalized diet plan without an energy restriction. The energy content of the diets was determined by a dietitian from the daily energy requirement of each participant. Participants met an assigned dietitian every two weeks during phase 1 and every four weeks during phase 2. During the entire protocol (both phase 1 and phase 2), either LPR or placebo supplementation was administered. A two-week washout period eliminating probiotic-containing products in the daily diet was also controlled for, prior to treatment initiation. The two supplements were administered orally. All participants ingested one capsule 30 min before breakfast and one capsule 30 min before dinner, on a daily basis. Participants were tested at baseline, week 12 (after the weight loss, phase 1), and week 24 (after the weight maintenance, phase 2) of the program. Compliance to the diet plan was assessed by comparing the prescribed diet composition (total daily energy intake and macronutrient composition) with the actual diet composition, measured every 2 weeks using a 24 h dietary recall. Compliance to the supplementation was measured using the compliance journal every two and four weeks during the weight loss and weight maintenance periods, respectively. The present study was conducted according to the guidelines in the Declaration of Helsinki. All procedures involving human participants were approved by the Ethics Committee of the Institut Universitaire de Cardiologie et de Pneumologie de Québec (CER: 20–449) and by Health Canada (144245), and the clinical trial number is NCT01106924. Written informed consent was obtained from all participants prior to the start of the study. Further details about the protocol were recently reported [20].

### 2.2. Participants

Participants were recruited through different forms of media in the Quebec City area, on the basis of the following inclusion and exclusion criteria: men and women between 18 and 55 years of age; absence of pregnancy, breastfeeding, or menopause (determined by the cessation of menstruation); stable body weight (body weight change <5 kg for three months before screening); body mass index (BMI) between 29 and 41 kg/m^2^, without associated co-morbidities.

### 2.3. Treatments

As previously described, the LPR capsules provided an average of 1.62 108 CFU Lactobacillus rhamnosus CGMCC1.3724 (LPR) during the study [20]. Participants received two capsules per day, corresponding to an average of 3.24 108 CFU/day. The LPR capsules contained a formulation consisting of 10 mg of a Lactobacillus rhamnosus CGMCC1.3724 powder, 300 mg of a mix of oligofructose and inulin (70/30; *v*/*v*), and 3 mg of magnesium stearate. The addition of prebiotics to the formulation allowed for a better resistance of probiotics to gut conditions [20]. The placebo capsules were the same colour and size as the LPR and contained 250 mg of maltodextrin and 3 mg of magnesium stearate. The stability of this strain has been assessed, as previously described in Sanchez et al. [20].

### 2.4. Measurements

As indicated above, measurements were performed before the study, and after both the weight loss (Phase 1) and weight maintenance (Phase 2) periods.

### 2.5. Appetite Sensations

A standardized breakfast test meal with 1 h post-meal appetite measurements was used to evaluate appetite sensations. To perform this test, participants were asked to arrive at the laboratory in the morning after a 12 h overnight fast, and to refrain from alcohol consumption and intense physical activity during the 24 h period before the testing session. A standardized breakfast was served between 7:30 a.m. and 8:30 a.m., in order to replicate the usual breakfast time of each participant. The energy content of the test meal was 733 kcal (3066 kJ) for men and 599 kcal (2504 kJ) for women [21]. All participants were instructed to consume all of the food within 30 min. Before, immediately after, and every 10 min for a one-hour period after the standardized breakfast, participants were asked to record their appetite sensations using Visual Analogue Scales (VAS) adapted from Blundell and Hill [22], that assessed their “desire to eat”, “hunger”, “fullness”, and “prospective food consumption”. Participants were asked to indicate on a scale from 0 to 150 mm, how they felt at the moment they answered these questions, e.g., “How strong is your desire to eat?” (very weak—very strong). Participants were also asked to rate the palatability of the breakfast using VAS.

Baseline appetite ratings immediately before the fixed breakfast test meal were referred to as fasting appetite sensations. The satiety efficiency was assessed using the satiety quotient (SQ) concept adapted from Green et al. [23]. The SQ (the extent to which a standard portion of food can reduce subjective appetite sensations) was calculated from VAS, in response to a fixed-calorie meal. Thus, for each appetite sensation (AS), the SQ value was calculated using the following equation:

(1) $$\mathrm{SQ}\;\left(\mathrm{mm}/100\;\mathrm{kcal}\right)=\frac{\left(\mathrm{fasting}\;\mathrm{AS}\;-\;\mathrm{mean}\;60\;\min\;\mathrm{post}-\mathrm{meal}\;\mathrm{AS}\right)\;\times \;100}{\mathrm{energy}\;\mathrm{content}\;\mathrm{of}\;\mathrm{the}\;\mathrm{test}\;\mathrm{meal}\;\left(\mathrm{kcal}\right)}$$

Because the energy content of the fixed meal was different for men and women, the theoretical possible range of SQ values was between −20 and 20 for men and −25 and 25 for women, with a higher SQ representing greater satiety and a lower SQ indicating lower satiety. The SQ is considered to be a valid indicator of satiety because it takes into account pre-meal appetite sensations and considers the caloric content of the meal. It has been shown to be positively associated with energy intake [21,24] and is a reliable marker.

### 2.6. Ad libitum Energy Intake

At lunch, each participant was provided with a buffet-type meal to measure ad libitum energy intake. The administration of a buffet-type meal in the laboratory was executed according to procedures previously described [25]. The buffet-type meal was served 3.5 h after the end of the breakfast. A cold buffet-type meal containing a variety of foods was offered, and participants were instructed to eat until they were “comfortably full”, over a 30 min period. In order to control for external stimuli that could affect appetite, participants were isolated in a quiet room with no sensory distractions. All foods were weighed to the nearest 0.1 g immediately before and after test meals. The same appetite measurements as for the breakfast test meal were completed before, immediately after, and at hourly intervals for a four-hour period after the buffet-type meal.

### 2.7. Daily Energy Intake and Physical Activity

A standardized paper-based three-day dietary record (two weekdays and one weekend day) [26] was obtained from each participant. This diary was completed at home, after the participants had received detailed instructions by a dietitian. A computerized version of the Canadian Nutrition File (version 2005) was used to enter the foods from the diaries in order to determine the macro- and micronutrient content of the diet, as well as the total daily energy intake [27]. An average of the three days was used for analyses. This measurement was repeated at the end of the weight loss (phase 1: Baseline to week 12) and weight maintenance (phase 2: Weeks 12 to 24) periods. A three-day physical activity record [28] was also completed at home on the same days as the completion of the dietary record, to assess the maintenance of physical activity habits over the course of the study protocol.

In addition, participants completed a 24 h dietary recall with the assistance of the dietitian every two weeks during phase 1 and every four weeks during phase 2. For each participant, these records or recalls provided reference information to the dietitian, in order to standardize the counselling and related guidelines over the two phases of the protocol.

### 2.8. Resting Metabolic Rate and Calculation of the Estimated Energy Deficit

As indicated above, each participant received a personalized diet plan targeting a 500 kcal/day (2092 kJ/day) energy restriction for the first 12 weeks of the program. During phase 2, each participant received a personalized diet plan without an energy restriction. The diet plan was based on an exchange food group list adapted from the Meal Planning for People with Diabetes [29]. Aside from the supplement, both the groups were limited to a consumption of a maximum of four servings of products supplemented with probiotics per week. A dietitian determined the energy content of the diets based on the daily energy requirement of each participant, which was estimated using resting energy expenditure (REE) and multiplying it by an activity factor based on the physical activity record. The REE was determined after a 12 h overnight fast in participants that had rested for at least 15 min in a standardized supine position. The REE was measured at the baseline and was re-assessed after the weight loss and the weight maintenance periods, by using indirect calorimetry [20].

### 2.9. Body Weight and Composition Measurements

Body weight was assessed at the baseline, every two weeks during phase 1, as well as at the end of this phase (week 12) of the protocol, and every four weeks during Phase 2 and at the end of this phase (week 24), using a digital scale (Tanita, Arlington Heights, IL, USA). Body fat and fat-free mass were measured by dual-energy X-ray absorptiometry (GE Medical Systems Lunar, Diegem, Belgium) at the beginning and at the end of the weight loss program and weight maintenance period.

### 2.10. Three-Factor Eating Questionnaire

The Three-Factor Eating Questionnaire (TFEQ) [30] measures three dimensions of eating behaviour including cognitive restraint, disinhibition, and hunger, with 51 items. Cognitive restraint is the intent to restrict the energy intake with the purpose of weight control. A higher cognitive restraint score represents a high restriction in energy intake to control body weight. Disinhibition is the susceptibility to overeat, whereas hunger measures the susceptibility to feelings of hunger. A higher disinhibition score represents a higher susceptibility to overeat. Susceptibility to hunger represents the ability to cope with the sensation of hunger. A higher hunger score represents a higher susceptibility to eat, in response to hunger.

### 2.11. State-Trait Food Cravings Questionnaire

The State-Trait Food Cravings Questionnaire-Trait (FCQ-T) [31] measures nine dimensions of food cravings with 39 items. The food craving is defined as an intense desire to consume a particular food or food type that is difficult to resist [32]. The FCQ-T measures stable dimensions of craving, including intention to eat, positive reinforcement, negative reinforcement, lack of control, preoccupation with food, feelings of hunger, negative effect, cue-dependent eating, and guilty feelings. A higher FCQ-T score represents a higher susceptibility for craving.

### 2.12. Mood-Related Questionnaires

Participants also completed additional questionnaires at the baseline and at the end of the program. The Beck Depression Inventory (BDI) [33] was used to measure the level of depression. The BDI contains 21 questions, with a higher score representing greater symptoms of depression. The Body Esteem Scale (BE) [34] was used to measure body esteem, which refers to the self-evaluation of one’s body appearance. The BE includes 23 items to which participants respond using a five-point Likert scale, anchored at each end by never and always. The higher the score, the higher the body esteem. The Binge Eating Scale (BES) [35] was used to measure the behavioral, affective, and attitudinal components of the experience of binge eating. It is a 16-item self-report questionnaire where higher scores are positively correlated with higher levels of binge eating. The Perceived Stress Scale (PSS) [36] was used to measure nonspecific perceived stress. It contains 10 questions and the higher the score is, the higher the perceived stress is. The State-Trait Anxiety Inventory (STAI) [37] was used to measure feelings of anxiety (State = now and Trait = in general). The STAI has 40 questions and higher scores on the State and Trait Scale indicate higher levels of anxiety.

### 2.13. Statistics

Statistical analyses were performed using SAS version 9.2 (SAS Institute Inc., Cary, NC, USA). The outcome measures were analyzed in the context of secondary analysis using analysis of covariance (ANCOVA), considering changes over time in a mixed model setting treatment as the independent variable, while correcting for baseline values in the model. The effect of treatment was examined for each sex. The analyses were performed for the intention-to-treat population. Statistical significance was set at 5% and no correction of the significance level was applied to adjust for multiple testing. The sample size was calculated using the statistical and power analysis software NCSS.

## 3. Results

### 3.1. Body Weight and Composition

As previously reported [20], the mean body weight loss exceeded 5 kg at the end of the 24-week protocol for both the LPR and control male participants and for the female participants receiving the LPR formulation. This was in contrast with the weight loss observed in the female control group whose mean weight loss was about half of that achieved by the other three groups. In each subgroup, body fat loss explained at least 77% of the weight loss over the 24-week program.

### 3.2. Baseline Data

One participant was excluded from the study due to poor compliance to the treatment. Baseline values for appetite sensations and eating behaviour traits are presented in Table 1. There were no differences between LPR and placebo groups for both sexes, except for the TFEQ hunger score, which was significantly greater in women receiving the LPR formulation than in their placebo counterparts. Furthermore, there were no differences between LPR and placebo groups for physical activity levels [38].

### 3.3. Response of Appetite Sensations to Food Intake

As shown in Table 2, the desire to eat in the fasting state increased more in the LPR group than in the placebo group for women after 24 weeks (changes in the desire to eat in the fasting state after the LPR formulation compared with the placebo: 15.8 ± 7.2; *p* = 0.03). In men, fullness in the fasting state was higher after the LPR formulation in Phase 2, when compared with the placebo (changes in fullness in the fasting state compared with placebo: 12.2 ± 5.0; *p* = 0.02). In both women and men, the SQ at the buffet for the desire to eat was higher in the LPR group than in the placebo group after Phase 1 (changes in the SQ at the buffet for the desire to eat compared with placebo: men, 2.6 ± 1.2; *p* = 0.03; women, 3.5 ± 1.5; *p* = 0.02) (Table 2). The same trend was observed for the changes in desire to eat at breakfast time; however, the differences were not statistically significant.

### 3.4. Energy Intake

As shown in Table 2, there was no difference between the LPR group and placebo for the reported energy intake (three-day dietary record) and the energy intake measured (ad libitum energy intake). Furthermore, there was no difference between the LPR group and placebo for the percentage of macronutrients in both the three-day dietary record and the ad libitum energy intake.

### 3.5. Eating Behaviour Traits

As shown in Table 3, cognitive restraint was lower in the LPR group compared to the placebo in women and men after 24 weeks (Phase 1 plus Phase 2) (changes in TFEQ cognitive restraint during Phase 1 compared with placebo in women: −0.9 ± −0.4; *p* = 0.02; changes in TFEQ cognitive restraint during Phase 1 plus Phase 2 compared with placebo in women: −2.0 ± −0.8; *p* = 0.01; changes in TFEQ cognitive restraint during Phase 1 compared with placebo in men: −1.5 ± 0.6; *p* = 0.01; changes in TFEQ cognitive restraint during Phase 1 plus Phase 2 compared with placebo in men: −3.4 ± 1.2; *p* = 0.05). Specifically, the increase in this trait was less pronounced for individuals taking the LPR supplementation.

The TFEQ also revealed a significant decrease in disinhibition during Phase 1 for women (changes in TFEQ disinhibition compared with placebo: −1.1 ± 0.6; *p* = 0.05). Additionally, the TFEQ showed a greater decrease in hunger sensations during Phase 1 and the entire program for women (changes in TFEQ hunger during Phase 1 compared with placebo: −2.1 ± 0.8; *p* = 0.007; changes in TFEQ hunger during Phase 1 plus Phase 2 compared with placebo: −1.7 ± 0.7; *p* = 0.02) (Table 3).

A beneficial treatment effect of the LPR supplementation was also found for the State-Trait Food Cravings Questionnaire-Trait (total score) during Phase 1 and the entire program (Phase 1 plus Phase 2) for women (changes in FCQ-T total score during Phase 1 compared with placebo: −10.6 ± 4.8; *p* = 0.03; changes in FCQ-T total score during Phase 1 plus Phase 2 compared with placebo: −10.8 ± 5.3; *p* = 0.05).

### 3.6. Mood-Related Factors

**Figure 1:**
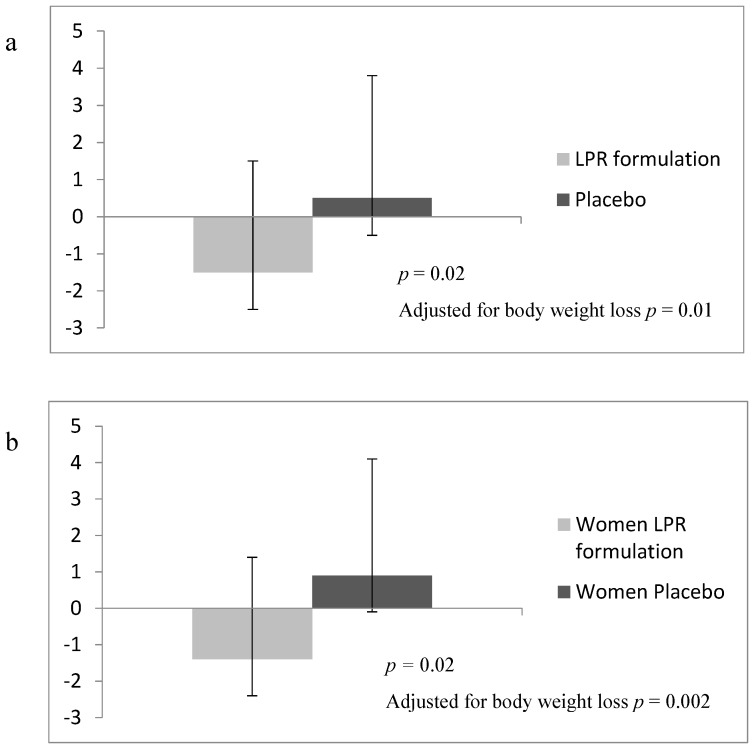
Variation in BDI scores between LPR formulation and placebo groups in (**a**) men and women and (**b**) women only. Values are means ± standard error of the mean. Results represent the variation of the BDI scores over the duration of the entire program.

As demonstrated in Table 3, the BE score was higher in the female LPR group compared to the placebo after Phase 2 (changes in BE score during Phase 2 compared with placebo: 3.8 ± 1.8; *p* = 0.04), and there was a trend towards a treatment effect for the BES during the entire program for women (changes in BE score during Phase 2 compared with placebo: 3.6 ± 1.9; *p* = 0.06). Table 3 also shows that the LPR supplementation induced a significant decrease in the BDI score, whether it was documented in the overall group or in the female subjects. This effect was in contrast with the small increase in BDI that was observed in the placebo group for the overall sample and the female group. Additionally, Figure 1 illustrates that these opposite changes in the supplemented and placebo groups were significantly different, even after statistical adjustment for body weight loss.

## 4. Discussion

The main hypothesis that the LPR formulation can positively influence appetite sensations, mood, and eating behavior traits in obese men and women under conditions of negative energy balance and subsequent body weight maintenance, was supported in this study. This was the first study performed in the context of nutritional supervision aimed at a decrease in energy intake. As indicated above, LPR-treated women displayed a higher mean body weight loss compared to women receiving the placebo. However, no effect of the LPR treatment on body weight was observed in men. The results of this study show some beneficial effects on mood and eating behaviour traits in women who achieved a greater body weight loss in the LPR formulation group, compared to participants receiving the placebo. Specifically, a lower BDI score, lower hunger sensations, and a lower disinhibition were observed in the LPR-treated women. Although this may also have been a reflection of the weight loss achieved, probiotics have been suggested to play a role in mood [37]. Beneficial effects of the LPR supplementation, such as an attenuation of the increase in cognitive restraint, were also observed in men.

Based on our previous research experience [16,39], we anticipated that the greater weight loss observed in the female LPR subgroup would have made the control of appetite more difficult. However, except for the increase in the fasting desire to eat in women, our results suggest that appetite control might have been facilitated by the LPR formulation in women. Specifically, the ad libitum meal revealed that the SQ for the desire to eat after Phase 1 in the LPR-treated women more significantly increased than in their placebo counterparts. This suggests that they displayed a greater efficiency to suppress the desire to eat after the buffet during the weight loss phase, compared to the placebo participants. The observation of the expected fasting desire to eat in supplemented individuals might be viewed as contradicting the main mechanistic hypothesis presented in the current study. However, the increase in the satiety efficiency observed after the buffet-type meal, leading to an adequate compensation in the increase in the fasting desire to eat, cannot be clearly determined. It is, however, relevant to consider that, based on their dietary records, supplemented women reported a more pronounced decrease in daily energy intakes compared to those on the placebo, although not to a statistically significant extent. Specifically, this difference in daily energy intake exceeded 100 kcal per day and thus provides some justification to further examine the potential impact of changes in fasting appetite sensations, as well as the satiety efficiency on the daily energy intake in the context of a weight-reducing program. The increase in the SQ for desire to eat is consistent with the greater decrease in the disinhibition score during Phase 1 of the LPR-treated women, when compared with the placebo-treated women. Since a high disinhibition is predictive of an increased appetite [24] and a greater risk of weight gain [1,21], we interpret the reducing effect of LPR formulation on disinhibition in women as an outcome that might have further contributed to an accentuation of weight loss over time. Moreover, we observed a decrease in the TFEQ hunger feeling during Phase 1 and the entire program in-LPR treated women, compared to placebo-treated women. We speculate that this effect was also in concordance with a better weight maintenance in LPR-treated women compared to the control group [20]. Taken together, these results suggest that appetite control was slightly different and may have been improved in LPR treated women in a context that would have normally made it more difficult to control appetite sensations due to their increased weight/fat loss [39].

A higher decrease in food cravings was also observed in the LPR- versus placebo-treated women during the weight loss and weight maintenance program. Recent studies show that an increase in food cravings has been associated with caloric restriction [40,41], suggesting that food craving can influence weight loss. On the other hand, some studies have demonstrated a decrease in cravings that was associated with weight loss [40,42,43,44]. As food cravings are suggested to be both an obstacle to weight loss and a promoter of regain after weight loss [45,46,47], we suggest that the decrease in food cravings reported here may help women to maintain weight loss over time.

Furthermore, the level of cognitive restraint was increased during the weight loss phase in the four groups of participants. These results were concordant with other studies, which have shown an increase in restraint during weight loss [48] and may contribute to the occurrence of resistance to further body weight loss over time. Accordingly, a high restriction, and a particularly high level of rigid restraint, has been associated with a higher BMI [49] and weight gain [50]. This increase in the level of cognitive restraint was less pronounced in men receiving the LPR formulation than in those of the placebo group. This observation is particularly interesting since both male groups achieved comparable weight loss during Phase 1. This suggests that if Phase 1 would have been extended, it may have ultimately made a difference in the ability to lose weight in response to the nutritional intervention. This perception is concordant with the increase in fasting fullness observed in supplemented men during the weight maintenance phase of the study.

A major challenge for health professionals in the field of obesity management is the ability to propose a weight reduction program that does not favor body weight regain. In general, the relapse of body weight in ex-obese individuals is primarily determined by changes in appetite that promote a significant increase in meal-time energy intake [16]. In this regard, our results emphasize a potential application for supplementation with Lactobacillus rhamnosus CGMCC1.3724, to facilitate appetite control in weight-reduced obese individuals who generally experience difficulty in maintaining body weight, especially if nothing is done to incorporate healthy lifestyle practices that can compensate for the metabolic vulnerability conferred by the body fat loss. This was particularly obvious in LPR women who displayed favorable adaptations in appetite sensations, mood, and eating behavior traits, despite experiencing a weight loss that would have predicted opposite effects [39]. In addition, the lower increase in cognitive restraint scores in LPR-treated men also suggests a facilitation of the control of eating behavior traits. Thus, the supplementation of some probiotics can be realistically added to the list of environmental factors contributing to body weight loss and maintenance, such as physical activity [51,52] and a high protein diet [53].

The BDI provided one of the main findings of the study. As shown in Table 3 and Figure 1, the group of supplemented women displayed a favorable decrease in the BDI, that contrasted with the small increase observed in the placebo group. This difference in trends was also seen in men, although, in contrast with women, the between-subgroup difference did not reach statistical significance. The increase in the BDI score observed in placebo-treated women is concordant with our recently reported results, showing that substantial weight loss may accentuate depressive symptoms in obese individuals [54]. In this regard, the fact that the opposite trend was seen in LPR-supplemented females having reached a greater weight loss supports the idea of a clinical benefit of the supplementation. This is also in agreement with the fact that the difference in BDI changes in the two groups of women remained statistically significant after adjusting for body weight loss. Interestingly, this potential benefit of LPR was accompanied by a significant increase of the BES, measured after both Phases 1 and 2 in women.

Evidence suggests that various mechanisms may favor a gut-brain connection relevant to obesity management [55,56]. In this regard, the results of the present study provide support to the hypothesis of a gut-brain axis. Significant alterations in the composition of the gut may result in changes in the release of various satiety hormones, such as glucagon-like peptide 1 (GLP-1) and peptide tyrosine tyrosine (PYY); however, although these hormones were not measured in this study, our previously reported results showed a role for leptin, particularly in women [20]. Even if the present study was statistically powered to detect differences in body weight as the primary outcome, it also documented concordant behavioural benefits in women subjected to the probiotic supplementation. While the present study did not examine the biological mechanisms responsible for the beneficial effects on mood, a number of hypotheses have been made, including an increase in plasma tryptophan concentrations leading to a facilitation of serotonin turnover in the brain, and an improvement in the epithelial barrier function leading to decreased intestinal permeability [57].

As indicated above, a small amount of prebiotic was used in the LPR formulation, to increase the survival of probiotics [58,59,60]. It may be argued that some of the documented effects on appetite sensations and eating behaviours may be partially attributed to prebiotics. However, as previously explained [20], experimental evidence suggests that the amount of prebiotic included in our formulation was too low to significantly influence feeding behaviours. This viewpoint is supported by recent results [61] showing that the supplementation of much larger daily doses of a comparable prebiotic formulation (short-chain oligofructose) was not sufficient to acutely influence appetite.

Figure 1 illustrates the global impact of the program on the BDI score. The treatment effect was particularly obvious in women for whom the program induced opposite effects, depending on the condition. As expected, a small increase was observed in the placebo group, whereas the BDI score decreased in the LPR group, despite their greater body weight loss. The same trend was found in men, although it was not statistically significant. It is to be noted that the difference in the BDI observed at the end of the program in women remained significant (*p* < 0.05) after statistical adjustments for body weight loss.

## 5. Conclusions

In summary, the results of this study show that women, having benefited from an accentuation of body weight loss in response to a diet-based supervision combined with LPR supplementation, also displayed concordant changes in satiety efficiency, eating behaviours, and mood. These changes included an increase in the SQ for desire to eat, a decrease in the disinhibition and hunger behaviours documented by the TFEQ, a more pronounced decrease in food cravings, and an increase in body esteem. Additionally, despite their greater weight loss, the LPR female subgroup displayed a favorable change in the BDI score that was significantly in contrast with that observed in the control group. Interestingly, men also benefited from the LPR supplementation, since the expected increase in cognitive restraint was lower than that of their control participants exhibiting the same weight loss. Thus, if we also consider that no detrimental effect of the LPR supplementation was found in the present study, it seems reasonable to conclude that this gut-targeted supplement can favorably influence eating and emotion-related behaviors. In conclusion, LPR supplementation improves appetite sensations, eating, and emotion-related behaviors, thus lending support to the hypothesis that the gut-brain axis may impact appetite control and obesity management.

## Figures and Tables

**Table 1:** Baseline characteristics, energy intake, appetite sensation markers, and questionnaires.

Variables	LPR formulation	Placebo	Men		Women	
	62	63	LPR Formulation (*N* = 24)	Placebo (*N* = 24)	LPR Formulation (*N* = 38)	Placebo (*N* = 39)
**Subjects characteristics**						
Age (years)	35.0 (10.0)	37.0 (10.0)	37.0 (10.0)	38.0 (10.0)	34.0 (10.0)	36.0 (10.0)
Body weight (kg)	95.1 (13.9)	94.0 (14.9)	104.3 (13.0)	103.4 (15.0)	89.3 (11.1)	88.2 (11.5)
BMI (kg/m^2^)	33.8 (3.3)	33.3 (3.2)	34.0 (2.8)	33.5 (3.3)	33.6 (3.6)	33.2 (3.2)
Fat mass (%)	40.82 (7.68)	40.08 (8.22)	34.8 (8.3)	32.8 (9.1)	40.8 (8.2)	40.2 (7.6)
**Energy intake reported (3-day dietary record)**						
Mean daily kcal	2510 (676)	2362 (611)	2898 (550)	2752 (479)	2247 (637)	2122 (562)
Proteins (%)	17.1 (2.6)	16.9 (2.4)	17.2 (2.3)	17.1 (2.6)	16.9 (2.7)	16.7 (2.4)
Carbohydrates (%)	46.4 (6.9)	47.2 (5.6)	46.3 (5.5)	46.6 (5.9)	46.5 (7.6)	47.6 (5.4)
Lipids (%)	33.7 (6.2)	34.1 (4.9)	33.5 (4.4)	34.5 (5.2)	33.9 (7.1)	33.8 (4.5)
Alcohol (%)	2.8 (2.9)	1.8 (2.7)	3.0 (2.7)	1.8 (2.6)	2.7 (3.1)	1.9 (2.8)
**Energy intake measured (buffet type meal)**						
Kcal	908.6 (437.3)	906 (424.5)	1017 (407)	1096 (503)	799 (419)	750 (279)
Proteins (%)	17.3 (3.4)	17.3 (5.3)	17.5 (3.0)	16.8 (5.8)	16.4 (3.7)	16.8 (5.3)
Carbohydrates (%)	42.8 (8.2)	44.7 (10.1)	45.2 (8.2)	46.9 (11.9)	41.7 (8.4)	43.8 (9.2)
Lipids (%)	39.9 (8.5)	38.0 (10.2)	37.3 (8.3)	36.3 (11.2)	41.9 (8.7)	39.4 (10.0)
**Appetite sensations in fasting state (mm)**						
Desire to eat	96.6 (31.7)	96.5 (33.0)	98.1 (28.9)	96.0 (34.7)	95.7 (33.7)	96.8 (32.4)
Fullness	24.0 (25.4)	24.0 (27.7)	27.4 (30.0)	33.9 (33.7)	21.8 (22.1)	17.9 (21.6)
**SQ at breakfast (mm/ kcal)**						
Desire to eat	10.2 (4.9)	10.4 (5.8)	8.6 (4.1)	6.8 (4.3)	11.2 (5.2)	12.7 (5.5)
Fullness	11.1 (5.6)	10.8 (7.4)	8.4 (5.0)	6.0 (4.6)	12.8 (5.4)	13.8 (7.3)
**SQ at buffet (mm/ kcal)**						
Desire to eat	10.3 (6.2)	10.2 (5.9)	7.1 (3.8)	7.7 (3.9)	12.5 (6.5)	11.6 (6.3)
Fullness	10.3 (5.6)	9.9 (5.9)	8.2 (3.7)	8.8 (4.0)	11.7 (6.3)	10.4 (6.7)
**Eating behaviour traits (TFEQ)**						
Cognitive restraint	8.3 (4.0)	8.3 (3.7)	8.0 (3.2)	7.3 (3.3)	8.4 (4.5)	8.9 (3.9)
Disinhibition	7.8 (3.0)	8.0 (3.1)	6.7 (3.2)	8.2 (3.2)	8.4 (2.8)	7.9 (3.0)
Hunger	6.3 (3.3)	6.2 (3.5)	5.6 (3.7)	8.2 (2.6)	6.7 (3.0) *	5.0 (3.5)
**State-Trait Food Cravings Questionnaire-Trait (FCQ-T)**						
Total score	108.8 (25.0)	110.3 (28.3)	100.8 (24.6)	111.3 (24.2)	114.4 (24.0)	109.8 (30.6)
**BECK Depression Inventory**						
Total score	4.4 (4.1)	4.7 (4.2)	3.6 (3.5)	3.6 (2.9)	4.96 (4.4)	5.2 (4.7)
**Body Esteem Scale (BE)**						
Total score	38.7 (10.9)	35.8 (10.8)	45.6 (11.3)	37.8 (11.4)	34.6 (8.4)	34.6 (10.4)
**Binge Eating Scale (BES)**						
Total score	12.0 (6.6)	12.2 (6.6)	10.0 (6.6)	12.8 (6.9)	13.4 (6.4)	11.8 (6.5)
**Perceived Stress Scale (EPS)**						
Total score	14.1 (6.0)	14.4 (6.0)	12.1 (5.8)	12.9 (5.7)	15.4 (5.9)	15.3 (6.1)
**Spielberger State-Trait Anxiety Inventory (State Anxiety)**						
Total score	28.9 (6.5)	28.2 (5.9)	29.8 (7.8)	27.4 (5.9)	28.3 (5.6)	28.7(5.9)
**Spielberger State-Trait Anxiety Inventory (Trait Anxiety)**						
Total score	37.3 (7.8)	37.0 (6.7)	35.8 (7.9)	37.9 (7.6)	38.3 (7.7)	36.5 (6.2)

Values for age, body weight, BMI and fat mass were previously reported [20]; * Means values were significantly different from those of the placebo group (*p* < 0.05).

**Table 2:** Changes in body weight, appetite sensations, and energy intake during the program.

Variables Changes	All participants		Men		Women	
	LPR Formulation	Placebo	LPR Formulation	Placebo	LPR formulation	Placebo
**Number of participants**						
∆ Phase 1-baseline	52	53	23	22	29	31
∆ Phase 2-Phase 1	45	48	19	20	26	28
∆ Phase 2-baseline	45	48	19	20	26	28
Body weight (kg) **						
∆ Phase 1-baseline	−4.2 (3.2)	−3.4 (2.9)	−4.0 (3.4)	−4.6 (3.2)	−4.4 (3.0) *	−2.6 (2.3)
∆ Phase 2-baseline	−5.3 (4.3)	−3.9 (4.2)	−5.4 (4.8)	−5.7 (4.5)	−5.2 (4.0) *	−2.5 (3.5)
**Fasting state** Desire to eat (mm)						
∆ Phase 1-baseline	9.3 (39.8)	3.6 (37.0)	0.2 (46.5)	−4.2 (33.3)	16.2 (33.0)	9.1 (39.0)
∆ Phase 2-Phase 1	6.9 (30.6)	9.3 (40.5)	7.9 (27.6)	23.9 (34.2)	6.3 (33.0)	-1.1 (42.0)
∆ Phase 2-baseline	16.3 (32.8)	13.5 (37.5)	8.0 (35.6)	22.9 (34.3)	22.2 (29.9) *	6.8 (38.9)
Fullness (mm)						
∆ Phase 1-baseline	−3.2 (27.8)	1.9 (25.0)	−3.3 (33.3)	3.1 (31.6)	−3.1 (23.4)	1.1 (19.6)
∆ Phase 2-Phase 1	1.9 (17.1)	−3.4 (19.1)	5.8 (17.0) *	−10.0 (18.1)	−0.9 (16.9)	1.3 (18.7)
∆ Phase 2-baseline	−1.1 (30.0)	−0.2 (22.4)	2.6 (39.2)	−3.1 (25.0)	−3.7 (21.9)	1.9 (20.6)
**SQ at breakfast** Desire to eat (mm/kcal)						
∆ Phase 1-baseline	1.2 (6.6)	0.7 (6.2)	−1.1 (5.9)	0.2 (4.6)	2.8 (6.8)	1.1 (7.2)
∆ Phase 2-Phase 1	1.1 (5.8)	0.7 (6.5)	0.8 (4.7)	2.5 (4.1)	1.3 (6.4)	−0.6 (7.6)
∆ Phase 2-baseline	2.3 (5.3)	1.4 (6.5)	0.6 (4.6)	3.0 (5.0)	3.5 (5.5)	0.2 (7.2)
Fullness (mm/kcal)						
∆ Phase 1-baseline	1.4 (5.1)	0.3 (5.0)	0.2 (5.0)	0.2 (4.1)	2.3 (5.1)	0.4 (5.7)
∆ Phase 2-Phase 1	−0.3 (4.0)	0.7 (4.4)	−0.5 (3.8)	1.1 (3.1)	−0.3 (4.2)	0.4 (5.2)
∆ Phase 2-baseline	1.5 (5.3)	1.1 (5.5)	0.6 (6.1)	1.2 (5.6)	2.1 (4.7)	1.1 (5.6)
**SQ at buffet** Desire to eat (mm/kcal)						
∆ Phase 1-baseline	1.6 (7.1) *	−0.5 (4.8)	1.5 (5.0)	−0.2 (4.2)	1.6 (8.2) *	-0.7 (5.3)
∆ Phase 2-Phase 1	−0.5 (6.2)	0.9 (5.9)	−0.6 (3.0)	0.3 (3.7)	−0.4 (7.6)	1.4 (7.4)
∆ Phase 2-baseline	1.0 (7.5)	−0.1 (6.5)	1.8 (5.0)	−1.1 (3.8)	0.6 (8.6)	0.6 (7.9)
Fullness (mm/kcal)						
∆ Phase 1-baseline	−22.0 (12.9)	−17.9 (9.0)	−16.4 (6.6)	−15.3 (6.4)	−25.4 (14.5)	-20.2 (10.4)
∆ Phase 2-Phase 1	0.9 (6.8)	−1.8 (5.1)	1.5 (3.0)	−0.5 (3.6)	0.6 (8.3)	−3.0 (6.0)
∆ Phase 2-baseline	−21.0 (11.6)	−18.8 (7.6)	−14.3 (6.0)	−15.0 (5.7)	−24.4 (12.3)	-21.9 (7.6)
**Energy intake reported (3-day dietary record)** Mean daily kcal						
∆ Phase 1-baseline	−426.3 (65.2)	405.6 (65.7)	−405 (454)	−435 (454)	-488 (505)	-375 (423)
∆ Phase 2-Phase 1	100.0 (67.0)	78.8 (67.5)	44 (619)	46 (431)	156 (275)	112 (438)
∆ Phase 2-baseline	−343.0 (68.0)	−323.2 (68.1)	−341 (432)	−375 (457)	−345 (455)	−272 (456)
**Energy intake reported (3-day dietary record)** Proteins (%)						
∆ Phase 1-baseline	2.2 (0.4)	2.5 (0.4)	0.6 (3.4)	1.7 (3.5)	3.7 (3.9)	3.4 (3.5)
∆ Phase 2-Phase 1	−0.1 (0.4)	0.1 (0.4)	0.7 (2.7)	0.5 (4.0)	−0.1 (3.7)	−0.5 (3.2)
∆ Phase 2-baseline	2.5 (0.5)	2.8 (0.5)	1.0 (3.8)	2.3 (4.1)	3.9 (3.6)	3.0 (2.5)
**Energy intake reported (3-day dietary record)** Carbohydrates (%)						
∆ Phase 1-baseline	3.2 (0.8)	2.1 (0.8)	4.5 (8.1)	1.8 (7.5)	2.6 (6.8)	1.6 (6.3)
∆ Phase 2-Phase 1	−1.6 (0.8)	−0.1 (0.8)	−3.9 (5.6)	1.6 (6.6) *	−0.5 (6.3)	−0.9(7.4)
∆ Phase 2-baseline	1.3 (0.8)	2.3 (0.8)	1.0 (8.0)	2.6 (7.7)	2.5 (8.3)	0.7 (5.5)
**Energy intake reported (3-day dietary record)** Lipids (%)						
∆ Phase 1-baseline	−4.6 (0.8)	−4.2 (0.8)	−3.8 (5.7)	−3.8 (6.4)	−5.4 (8.0)	−4.4 (6.6)
∆ Phase 2-Phase 1	1.8 (0.8)	0.2 (0.8)	2.7 (4.8)	−0.9 (6.4) *	1.2 (6.3)	0.8 (7.0)
∆ Phase 2-baseline	−2.9 (0.8)	−4.2 (0.8)	−1.3 (6.5)	−4.2 (8.1)	−4.7 (7.8)	−3.5 (4.9)
**Energy intake reported (3-day dietary record)** Alcohol (%)						
∆ Phase 1-baseline	−0.9 (0.3)	−0.3 0(0.3)	−1.3 (2.6)	0.3 (3.2) *	−1.0 .(2.1)	−0.6 (1.7)
∆ Phase 2-Phase 1	−0.1 (0.3)	−0.2 (0.3)	0.5 (2.4)	−1.2 (3.1)	−0.6 (3.0)	0.6 (3.1)
∆ Phase 2-baseline	−0.9 (0.3)	−0.9 (0.3)	−0.7 (3.3)	−0.8 (2.9)	−1.8 (3.4)	−0,3 (3.7)
**Energy intake measured (buffet type meal)** Mean daily kcal						
∆ Phase 1-baseline	−18.6 (39.2)	−35.2 (38.4)	−30 (464)	−62 (340)	−22 (179)	−39 (336)
∆ Phase 2-Phase 1	0.5 (68.1)	102.9 (68.1)	12 (235)	115 (194)	−16 (141)	62 (377)
∆ Phase 2-baseline	−38.5 (50.4)	35.0 (49.4)	−46 (583)	47(396)	−45 (197)	20 (413)
**Energy intake measured (buffet type meal)** Proteins (%)						
∆ Phase 1-baseline	0.6 (0.5)	0.5 (0.5)	−0.8 (3.0)	0.4 (3.1)	2.0 (4.0)	0.5 (5.5)
∆ Phase 2-Phase 1	0.7 (0.7)	−0.1 (0.7)	2.3 (4.9)	−0.3 (3.3)	−0.7 (5.3)	0.1 (5.2)
∆ Phase 2-baseline	1.4 (0.7)	0.5 (0.7)	1.6 (6.2)	0.2 (5.0)	1.5 (4.1)	0.4 (5.7)
**Energy intake measured (buffet type meal)** Carbohydrates (%)						
∆ Phase 1-baseline	0.1 (1.1)	2.9 (1.1)	−0.1 (7.7)	−0.1 (11.5)	1.5 (9.7)	4.1 (10.8)
∆ Phase 2-Phase 1	−3.7 (1.2)	−1.4 (1.1)	−7.0 (9.7) *	−1.2 (6.6)	0.5 (8.0)	−2.6 (8.9)
∆ Phase 2-baseline	−2.5 (1.3)	0.6 (1.3)	−5.8 (10.2)	−2.1 (12.6)	1.2 (9.9)	2.2 (9.4)
**Energy intake measured (buffet type meal)** Lipids (%)						
∆ Phase 1-baseline	−0.7 (1.2)	−3.3 (1.2)	0.8 (8.8)	−0.3 (10.4)	−3.5 (10.5)	−4.6 (10.5)
∆ Phase 2-Phase 1	2.9 (1.1)	1.6 (1.0)	4.7 (8.0)	1.5 (5.5)	0.2 (8.2)	2.5 (7.6)
∆ Phase 2-baseline	−1.1 (1.3)	−1.1 (1.2)	4.2 (9.0)	1.8 (11.5)	−2.7 (9.1)	−2.7(10.2)

* Means values were significantly different from those of the placebo group (*p* < 0.05); ** Body weight changes were previously reported in reference 20.

**Table 3:** Changes in the questionnaire during the program.

Variables Changes	All Participants		Men		Women	
	LPR Formulation	Placebo	LPR Formulation	Placebo	LPR Formulation	Placebo
**Number of Participants**						
∆ Phase 1-baseline	52	53	23	22	29	31
∆ Phase 2-Phase 1	45	48	19	20	26	28
∆ Phase 2-baseline	45	48	19	20	26	28
**Three-Factor Eating Questionnaire** Cognitive restraint						
∆ Phase 1-baseline	3.5 (3.5) *	4.8 (3.7)	3.0 (3.6) *	5.9 (3.8)	3.9 (3.5)	4.0 (3.5)
∆ Phase 2-Phase 1	0.1 (2.6)	0.0 (3.0)	−0.3 (1.9)	0.6 (2.8)	0.5 (3.1)	−0.5 (3.2)
∆ Phase 2-baseline	3.7 (4.4) *	5.3 (4.3)	2.6 (4.3) *	6.7 (4.5)	4.4 (4.4)	4.2 (4.0)
Disinhibition						
∆ Phase 1-baseline	−2.0 (2.0)	−1.3 (2.3)	−1.6 (1.8)	−1.5 (2.3)	−2.2 (2.1) *	−1.1 (2.3)
∆ Phase 2-Phase 1	0.3 (1.4)	−0.4 (1.9)	0.5 (1.4)	−0.4 (1.6)	0.2 (1.4)	−0.5 (2.1)
∆ Phase 2-baseline	−1.7 (2.3)	−1.8 (2.1)	−1.2 (2.5)	−1.7 (2.1)	−2.1 (2.2)	−1.8 (2.2)
Hunger						
∆ Phase 1-baseline	−2.6 (3.0)	−1.8 (2.7)	−1.8 (3.5)	−2.9 (3.0)	−3.2 (2.6) *	−1.1 (2.2)
∆ Phase 2-Phase 1	0.3 (2.0)	−0.2 (2.7)	0.3 (1.7)	−0.3 (2.9)	0.4 (2.2)	−0.0(2.5)
∆ Phase 2-baseline	−2.4 (2.4)	−2.0 (2.7)	−1.8 (3.1)	−3.3 (2.5)	−2.8 (1.8) *	−1.0 (2.5)
**State-Trait Food Cravings Questionnaire-Trait (FCQ-T)**						
∆ Phase 1-baseline	−16.1 (15.6)	−8.1 (18.0)	−11.5 (9.7)	−6.8 (21.7)	−19.5 (18.2) *	−8.9 (15.2)
∆ Phase 2-Phase 1	−3.1 (10.4)	−4.1 (11.9)	−3.6 (9.7)	−5.1 (9.8)	−2.8 (11.1)	−3.4 (13.5)
∆ Phase 2-baseline	−19.2 (18.8)	−13.2 (18.2)	−13.8 (14.6)	−14.5 (22.7)	−23.2 (20.8) *	−12.4 (14.8)
**BECK Depression Inventory**						
∆ Phase 1-baseline	0.1 (2.9)	−0.6 (3.1)	0.5 (2.7)	−0.1 (2.5)	−0.2 (3.1)	−1.0 (3.5)
∆ Phase 2-Phase 1	−1.3 (2.7) *	0.6 (3.2)	−2.0 (3.1)	−0.2 (3.2)	−0.8 (2.3) *	1.4 (3.1)
∆ Phase 2-baseline	−1.5 (3.0) *	0.5 (3.3)	−1.5 (3.3)	−0.1 (3.6)	−1.4 (2.8) *	0.9 (3.2)
**Body Esteem Scale (BE)**						
∆ Phase 1-baseline	6.2 (6.7)	6.6 (6.9)	4.2 (5.7)	6.5 (7.1)	7.4 (7.1)	6.7 (6.9)
∆ Phase 2-Phase 1	4.2 (7.4)	3.0 (6.4)	3.1 (7.7)	6.2 (4.9)	4.8 (7.3) *	0.7 (6.4)
∆ Phase 2-baseline	9.8 (7.7)	10.2 (6.3)	7.9 (8.3)	14.3 (5.0)	11.0 (7.2) †	7.3 (6.4)
**Binge Eating Scale (BES)**						
∆ Phase 1-baseline	−4.0 (4.5)	−3.6 (4.7)	−2.5 (3.9)	−2.6 (5.0)	−5.2 (4.7)	−4.3 (4.6)
∆ Phase 2-Phase 1	−0.5 (2.6)	−0.7 (3.3)	−0.6 (2.8)	−0.4 (3.1)	−0.5 (2.5)	−1.0 (3.4)
∆ Phase 2-baseline	−3.8 (3.3)	−4.3 (4.7)	−2.8 (3.6)	−3.6 (5.5)	−4.5 (30.)	−4.7 (4.2)
**Perceived Stress Scale (PSS)**						
∆ Phase 1-baseline	−1.3 (5.2)	−1.2 (3.8)	−0.4 (4.4)	−0.5 (3.9)	−1.9 (5.7)	−1.6 (3.7)
∆ Phase 2-Phase 1	−0.0 (4.5)	0.2 (5.0)	−0.4 (4.0)	−0.4 (5.1)	0.2 (4.9)	0.7 (5.0)
∆ Phase 2-baseline	−1.6 (6.0)	−1.0 (4.6)	−1.5 (4.7)	−1.3 (4.8)	−1.7 (6.9)	−0.8 (4.5)
**State-Trait Anxiety Inventory (State Anxiety)**						
∆ Phase 1-baseline	0.2 (7.6)	−0.0 (7.5)	−0.4 (7.5)	1.9 (7.3)	0.6 (7.8)	−1.4 (7.6)
∆ Phase 2-Phase 1	0.4 (7.8)	1.7 (6.7)	−1.0 (6.2)	0.3 (6.6)	1.3 (8.7)	2.7 (6.8)
∆ Phase 2-baseline	−0.6 (8.1)	1.5 (8.9)	−1.7 (5.7)	2.7 (10.7)	0.1 (9.4)	0.7 (7.4)
**State-Trait Anxiety Inventory (Trait Anxiety)**						
∆ Phase 1-baseline	−0.9 (5.4)	−1.8 (4.0)	−0.5 (3.6)	−2.4 (4.4)	−1.2 (6.4)	−1.5 (3.8)
∆ Phase 2-Phase 1	−1.1 (4.2)	0.5 (5.2)	−1.4 (4.1)	−0.8 (4.6)	−1.0 (4.3) †	1.6 (5.4)
∆ Phase 2-baseline	−2.7 (5.4)	−1.4 (5.8)	−2.6 (3.8)	−2.9 (5.0)	−2.8 (6.3)	−0.5 (6.2)

* Means values were significantly different from those of the placebo group (*p* < 0.05); † Means values were different from those of the placebo group (*p* < 0.07).
